# A Network Approach to Studying the Associations Between Posttraumatic Stress Disorder Symptoms and Dissociative Experiences

**DOI:** 10.1002/jts.22488

**Published:** 2020-02-22

**Authors:** Angélique O. J. Cramer, IJsbrand Leertouwer, R. Lanius, Paul Frewen

**Affiliations:** ^1^ Department of Methodology and Statistics School of Social and Behavioral Sciences Tilburg University Tilburg the Netherlands; ^2^ Department of Psychiatry Western University London Ontario Canada

## Abstract

In recent years, there has been a growing recognition of a dissociative subtype of posttraumatic stress disorder (D‐PTSD), characterized by experiences of depersonalization (DP) and derealization (DR), among individuals with PTSD. Little is known, however, about how experiences of DP and/or DR are associated with the experience of other PTSD symptoms. The central aim of the present paper was to explore the associations among DP, DR, and other PTSD symptoms by means of a network analysis of cross‐sectional data for 557 participants whose overall self‐reported PTSD symptom severity warranted a probable PTSD diagnosis. Three notable findings emerged: (a) a strong association between DP and DR, (b) the identification of DP as the most central symptom in the network, and (c) the discovery that clusters of symptoms in the network were roughly consistent with *DSM‐5* PTSD criteria. We discuss these findings in light of some considerations, including the nature of our sample and the limits of interpreting cross‐sectional network models.

In recent years, there has been a growing recognition of a dissociative subtype of posttraumatic stress disorder (D‐PTSD) among individuals with PTSD. Such individuals report experiencing not only the core PTSD criteria of reexperiencing, avoidance, negative alterations of cognition and mood (NACM), and hyperarousal but also trauma‐related experiences of depersonalization (DP) and/or derealization (DR; Lanius, Brand, Vermetten, Frewen & Spiegel, 2012). For example, following the seminal paper by Wolf et al. (2012), Hanse, Ross, and Armour's (2017) systematic review of 11 latent class and profile analyses concluded that D‐PTSD could be reliably identified as a minority subgroup comprising approximately between one in every 10 to one in every three individuals with PTSD, depending on sample characteristics. In addition, the World Mental Health Survey (*N* = 25,018) identified a 12‐month D‐PTSD prevalence rate of approximately 14% (Stein et al., 2013). Neuroimaging studies have also differentiated people with D‐PTSD from those with PTSD who do not experience DP or DR during both a resting state and in response to trauma‐related stimuli (e.g., Harricharan et al., 2016, Lanius et al., 2010; Nicholson et al., 2017, 2016; Olivé et al., 2018).

Surprisingly, however, little is known about how DP and DR symptoms relate to the experience of other PTSD symptoms. A factor analysis of the criteria outlined in the fifth edition of the *Diagnostic and Statistical Manual of Mental Disorders* (*DSM‐5*) in a general population sample showed that a DP/DR latent variable also exhibited moderate loadings with risk‐taking (self‐destructive and/or reckless behavior; i.e., Criterion E2) and psychogenic amnesia (i.e., Criterion D1) symptoms (Frewen, Brown, Steuwe, & Lanius, 2015), and both Frewen et al. (2015) and, more recently, Ross, Banik, Dědová, Mikulášková, and Armour (2018) observed that individuals attributed to a latent D‐PTSD class were among those most likely to endorse the E2 and D1 Criteria, suggesting the potentially dissociative character of risk taking and amnesia regarding traumatic events. Further, Steuwe, Lanius, and Frewen (2012) found an equivalent fit for factor models specifying flashbacks of traumatic memories (*DSM‐5* Criterion B3) as loading onto either reexperiencing or dissociative factors, which is consistent with the regarding of flashbacks as a dissociative experience.

A relatively novel and rapidly growing field constitutes the network perspective on mental disorders (Borsboom & Cramer, 2013; Cramer & Borsboom, 2015; Cramer, Waldorp, van der Maas & Borsboom, 2010). From that perspective, a mental disorder is the potential consequence of symptoms that directly interact with one another in a network structure; for example, having nightmares leads to sleep problems, which leads to irritability, which, in turn, leads to self‐destructive behavior (Borsboom, 2017; Cramer et al., 2016). Network methodology allows for the estimation of network structures for, among other types of data, that which is cross‐sectional. Network analyses are increasingly reported in the empirical literature for multiple clinical constructs (see Fried et al., 2017, for a review), including PTSD (e.g., Afzali et al., 2017; Armour, Fried, Deserno, Tsai, & Pietrzak, 2017; de Schryver, Vindevogel, Rasmussen & Cramer, 2015; Fried et al., 2018; Knefel, Tran & Lueger‐Schuster, 2016; McNally et al., 2015). Taken together, these studies reveal a clustering of symptoms that is broadly similar to the *DSM‐5* criteria for PTSD symptomatology together with the observed generally poor fit of trauma‐related amnesia (see reviews by Armour, Fried, & Olff, 2017, and Armour, Müllerová & Elhai, 2016).

However, due to the fact that few standard measures of PTSD symptoms assess for DP and DR, the network connections between D‐PTSD symptoms and other PTSD symptoms remain largely unknown. Two recent papers of which we are aware have featured a network analysis of PTSD symptoms inclusive of DP and DR (Knefel et al., 2016; McBride, Hyland, Murphy, & Elklit, 2020). Knefel et al. (2016) assessed PTSD as determined by the *International Classification of Diseases* (11th rev.) complex PTSD (CPTSD) and borderline personality disorder (BPD) symptoms in 219 adults who experienced childhood trauma during foster care, approximately half of whom reported presently experiencing symptoms of either DP or DR. The authors observed that DP symptoms were highly central in the network, which may suggest that experiences of DP significantly affect, and are in turn affected by, other PTSD symptoms. As their study was not a focused network investigation of DP and DR symptoms, however, the particular PTSD symptoms with which DP and DR were most strongly connected were not discussed. In addition, given that obtained network structures are known to be influenced by the nodes that are and are not included (i.e., the effect of “missing nodes;” Fried & Cramer, 2017), it is unclear whether their findings would replicate in a network comprising *DSM‐5* PTSD symptoms rather than one composed of *ICD‐11* PTSD, CPTSD, and BPD symptoms. McBride and colleagues (2020) also recently investigated the connectivity of DP and DR by assessing *DSM‐5* PTSD symptoms in a sample of Danish adult survivors of childhood sexual abuse. Their network structure revealed a strong connection between DP and DR and, for example, a strong connection between DP and flashbacks. A limitation of the study, however, was that *DSM‐5* symptoms were measured across different questionnaires and response scales, introducing possible method bias.

The goal of the present research was to use network analysis to further investigate the connectivity of the D‐PTSD symptoms DP and DR with other *DSM‐5* PTSD symptoms, as measured on the same scale. In contrast to factor analysis, in which the covariance structure of a set of variables is explained by introducing one or more latent factors that have an, at best, unclear ontological stance (see Borsboom, Cramer, & Kalis, 2019), a network approach allows researchers to investigate direct associations between symptoms while controlling for all other symptoms—that is, “conditional (in)dependence relations.” As such, researchers are able to map symptom–symptom associations that may be indicative of actual causal relations and to statistically test for differences in the magnitude of these associations; for example, this approach allows researchers to assess whether the connection between DP and flashbacks is significantly stronger than the connection between DR and flashbacks. Due to the absence of a body of earlier empirical work on the subject matter and the fact that current network methods are data‐driven and exploratory, the present research is exploratory in nature. Analyses were conducted on 557 participants, previously described by Frewen et al. (2015), whose overall self‐reported PTSD symptom severity was suggestive of a probable *DSM‐5* PTSD diagnosis and of whom approximately one‐quarter to one‐third had probable D‐PTSD, depending on classification rules.

## Method

### Participants and Procedure

Institutional research ethics board approval was received from Western University. A community sample (*N* = 2,728) was recruited across three waves of data collection using Amazon's Mechanical Turk (MTurk; Amazon, Seattle, WA) web service, as described in a prior publication (Frewen et al., 2015). This service has been shown to be a valid recruitment strategy for mental health research (Chandler & Shapiro, 2016). Participants volunteered after reading a brief advertisement of the study and received a nominal compensation, via registration of their unique MTurk username, for the time required to complete the study. In the present study, we investigated a subsample of 557 participants who scored at or above a 38 on the PTSD Checklist for *DSM‐5* (PCL‐5; Weathers et al., 2013), which was, at that time, the recommended cutoff score for a probable PTSD diagnosis; this score threshold served as the a priori study inclusion criterion.

The sample used for the present study (*N* = 557) consisted mostly of female participants (70.9%) who were generally of middle age (*M* = 33.1 years, *SD* = 10.8) and identified as Caucasian (73.9%; for further details, see Frewen et al., 2015, and the Supplemental Materials for descriptive statistics on the measures for used to assess childhood trauma exposure). The majority of participants (63%) reported suffering from a diagnosed psychological problem either currently or in the past. The remaining participants either denied any history of diagnosed psychological disorders (33.2%) or declined to comment (4.3%). Participants completed the previously described measures in addition to surveys regarding childhood trauma history, other dissociative experiences, and general distress, all of which have been in a previous publication (Frewen et al., 2015).

### Measures

#### PTSD symptoms

The PCL‐5 (Weathers et al., 2013) is a 20‐item self‐report questionnaire in which each item corresponds to a *DSM‐5* PTSD symptom (e.g., “In the past month, how much were you bothered by repeated, disturbing, and unwanted memories of the stressful experience?”; Weathers et al., 2013). For each item, participants were asked to report on the past‐month frequency of a symptom, using a 5‐point Likert scale ranging from 0 (*not at all*) to 4 (*extremely*). The total score ranges from 0 to 80, with higher scores indicative of more severe PTSD symptomatology. A score of 38 is recommended as a cutoff, above which someone is more likely to qualify for a PTSD diagnosis (Weathers et al., 2013). The reliability of the PCL‐5 was adequate in the current sample, Cronbach's α = .76.

#### Depersonalization and derealization

The Trauma‐Related Altered States of Consciousness Questionnaire (TRASC‐Q; Frewen & Lanius, 2014, 2015) is a 10‐item self‐report questionnaire that aims to tap into the broad domain of dissociative experiences relevant to assessing D‐PTSD. The phrasing of the 10 items was developed based on feedback from clinicians and researchers with expertise in PTSD and dissociative disorders as well as from patients who attend a psychological trauma clinical research service with which the last two authors are affiliated. The TRASC‐Q demonstrated convergent validity with other measures of dissociative experiences (Frewen, Brown, & Lanius, 2017). For the purposes of the present network analysis, we were particularly interested in the connections exhibited by two of these 10 items, which are directly relevant to the assessment of D‐PTSD: (a) DP: “Out of Body Experience—feeling detached or separated from your body, for example, feeling like you are looking down on yourself from above, or like you are an outside observer of your own body”; and (b) DR: “Feeling like what you are experiencing is not real—a change in the way you perceive or experience the world or other people, so that things seem dreamlike, strange, or unreal.” Both items inquire about past‐month frequency of the experiences, and responses are scored on a 5‐point Likert scale that ranges from 0 (*not at all*) to 4 (*extremely*).

### Data Analysis

We performed two main analyses. First, we estimated a network structure for our data and investigated the accuracy of the estimations (*bootnet* package for *R*; Epskamp, Borsboom, & Fried, 2018). Second, we computed centrality measures (*qgraph* package for *R*) and investigated their stability (*bootnet* package for *R*). All visualizations were generated with the *qgraph* package for *R* (Epskamp, Cramer, Waldorp, Schmittmann, & Borsboom, 2012).

#### Network estimation and accuracy

We used current, state‐of‐the‐art methodology for estimating a network structure for ordinal or continuous data: A Gaussian graphical model (GGM; Lauritzen, 1996). A GGM is a network model in which the connections (i.e., edges) between variables represent estimates of partial correlations. As such, a connection, or edge, in a GGM can be interpreted as a conditional dependence relation: If any two variables are connected in a GGM, they are dependent after controlling for all other variables in the network. If two variables are not connected, they are independent given all other variables in the network.

In order to minimize the risk of false positives (i.e., a nonzero connection estimated although there is no actual connection), we estimated a GGM with a regularization technique called graphical least absolute shrinkage and selection operator (LASSO; Friedman, Hastie, & Tibshirani, 2008). With this technique, one adds a tuning parameter lambda (λ) that results in denser networks for low values and sparser networks with fewer edges for higher values. Because the “true” network structure is unknown (i.e., is the “true” network dense or sparse?), the procedure involves estimating network structures for various lambda values. The final model is chosen by using the extended Bayesian information criterion (BIC; see Epskamp & Fried, 2017, for more details). The result is a sparse, conservative network: The connections that are estimated to be present (i.e., nonzero) are likely true positives, whereas the connections that are estimated to be absent (i.e., zero) may be false negatives.

We used a bootstrapping procedure to further investigate the accuracy of edge weight estimations (Epskamp et al., 2018). Specifically, we bootstrapped 95% confidence intervals around each edge weight by means of randomly drawing with replacement 1,000 samples of the same size as the original sample, each time reestimating the network structure. This resulted in a sampling distribution for each edge weight from which the ½*α − 1–½ *α interval was used as a confidence interval (Cronbach's α = .05). In addition, we tested whether nonzero edges differed significantly from one another.

#### Centrality measures and stability

For the estimated network structure, we computed three centrality measures that indicate how interconnected a node is in a network, relative to other nodes: (a) node strength: the sum of all edge weights that connect a certain node to other nodes, (b) betweenness: the number of times a certain node lies on the shortest path between two other nodes, and (c) closeness: the inverse of the summed length of all shortest edges between a given node and all other nodes.

We investigated the stability of the ordering within each centrality measure (e.g., Node 1 has the highest degree of centrality, then Node 4, and so on) by means of a subsetting procedure (Epskamp et al., 2018). We randomly dropped participants from the sample (10%, 20%, through 90%) and recomputed centrality estimates. Based on this procedure, we estimated the correlation–stability coefficient (range: 0–1), with a value greater than .25 indicating moderate stability and a value greater than .5 indicating strong stability), which quantifies the maximum proportion of participants that can be dropped to retain, with 95% certainty, a correlation with the centrality measure from the original sample of higher than .70. Consequently, we only interpreted centrality measures with at least moderate stability. In addition, for interpretable centrality measures, we investigated whether symptoms differed significantly in terms of their centrality estimates. As sample size increases, such differences between centrality estimates become easier to detect, and the ordering of centrality estimates becomes more stable.

## Results

### Descriptive Statistics

Table 1 displays the means and standard deviations for each of the 20 PCL‐5 PTSD and the two D‐PTSD symptoms. Symptom means ranged between 1.15, for DP, and 3.08, for detachment. As could be expected given a subsample with a total score of at least 38 on the PCL‐5, the mean values for PTSD symptoms were somewhat elevated given the 0–4 response scale: The minimum mean score was for self‐destructive or reckless behavior at 1.41 and the maximum for detachment at 3.08. As a result, there was some indication of a ceiling effect, with a Spearman correlation between means and standard deviations of −.82. Some symptoms showed considerable variability, with relatively high standard deviations compared to their means, such as trauma‐related amnesia (*M* = 1.73, *SD* = 1.40), self‐destructive or reckless behavior (*M* = 1.41, *SD* = 1.36), and DP (*M* = 1.15, *SD =* 1.32).

**Table 1:** Means and Standard Deviations for Posttraumatic Stress Disorder Checklist for DSM‐5 (PCL‐5) and Dissociative‐Subtype Posttraumatic Stress Disorder (D‐PTSD) Symptoms

	Cluster and Symptom Number	Symptom	*Mean*	*SD*	Strength
1	B1	Intrusive thoughts	2.48	1.03	0.65
2	B2	Nightmares	2.01	1.30	−0.09
3	B3	Flashbacks	1.98	1.21	−0.16
4	B4	Emotional cue reactivity	2.85	0.98	0.80
5	B5	Physiological cue reactivity	2.46	1.14	0.56
6	C1	Avoidance of thoughts	2.67	1.05	−0.29
7	C2	Avoidance of reminders	2.64	1.10	0.63
8	D1	Trauma‐related amnesia	1.73	1.40	−1.97
9	D2	Negative beliefs	2.97	1.10	−0.28
10	D3	Blame of self or others	2.95	1.09	−0.07
11	D4	Negative trauma‐related emotions	3.07	0.92	0.89
12	D5	Loss of interest	2.76	1.10	0.31
13	D6	Detachment	3.08	0.98	0.93
14	D7	Restricted affect	2.63	1.17	−0.04
15	E1	Irritability/anger	2.53	1.16	−0.78
16	E2	Self‐destructive/reckless behavior	1.41	1.36	−1.20
17	E3	Hypervigilance	2.38	1.21	−0.65
18	E4	Exaggerated startle response	2.33	1.22	0.89
19	E5	Difficulty concentrating	2.68	1.10	−0.77
20	E6	Sleep disturbance	2.84	1.22	−2.03
21	DP	Depersonalization (TRASC‐Q)	1.15	1.32	2.28
22	DR	Derealization (TRASC‐Q)	1.74	1.36	0.41

*Note*. DP = depersionalization; DR = derealization; TRASC = Trauma‐Related Altered States of Consciousness Questionnaire. Symptom clusters and numbers refer to clusters in the *Diagnostic and Statistical Manual of Mental Disorders* (5th ed.), except the DP and DR symptoms, which are part of the TRASC‐Q.

### Network Estimation and Accuracy

**Figure 1:**
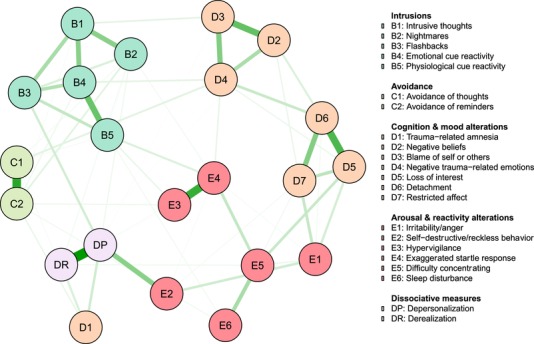
Regularized partial correlation network. Each node represents either one of 20 posttraumatic stress disorder (PTSD) symptoms as measured with the PTSD Checklist for *DSM‐5* (PCL‐5; node labels for this group start with either a B, C, D or E, each letter referring to the respective PTSD criterion in the fifth edition of the *Diagnostic and Statistical Manual of Mental Disorders* [*DSM‐5*]) or one of the two dissociative‐subtype PTSD (D‐PTSD) symptoms (node labels are DP and DR). Green edges indicate positive associations, and red edges indicate negative associations. Edge thickness represents the degree of association: The thicker the edge, the stronger the association. The position of the nodes in the network is based on the Fruchterman‐Reingold algorithm (Fruchterman & Reingold, 1991), which causes strongly associated symptoms to cluster in the middle. Symptoms with weaker associations are placed more towards the periphery of the figure. For the sake of clarity, only partial correlations larger than .03 are displayed in this figure.

**Figure 2:**
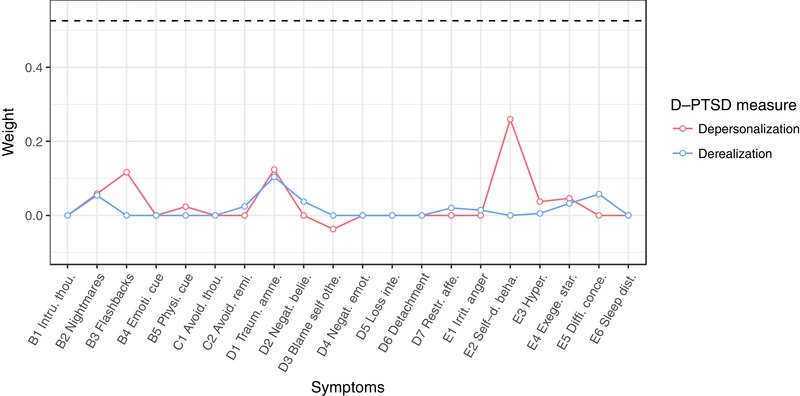
Edge weight estimates between either depersonalization (DP; red line) or derealization (DR; blue line) and PTSD Checklist for *DSM‐5* (PCL‐5) symptoms. The dashed line represents the edge weight between DP and DR. PTSD = posttraumatic stress disorder; *DSM‐5* = *Diagnostic and Statistical Manual of Mental Disorders* (5th ed.); D‐PTSD = dissociative‐subtype PTSD. Intru. thou. = intrusive thoughts; Emoti. cue = emotional cue reactivity; Physi. cue = physical cue reactivity; Avoid. thou. = avoidance of thoughts; Avoid remi. = avoidance of reminders; Traum. amne. = trauma‐related amnesia; Negat. belie. = Negative beliefs; Blame self othe. = Blame of self or others; Negat. emot. = negative trauma‐related emotions; Loss inte. = loss of interest; Restr. affe. = restricted affect; Irrit. anger = irritability/anger; Self‐d. beha. = self‐destructive/reckless behavior; Hyper. = hypervigilance; Exege. star. = exaggerated startle response; Diff. conce. = difficulty concentrating; Sleep dist. = sleep disturbance.

The estimated network structure is visualized in Figure 1. The stability analyses indicated that the network structure was accurately estimated with small‐to‐moderate confidence intervals (see Supplementary Materials for plots visualizing bootstrapped confidence intervals and significance tests for nonzero edge weight differences). Regarding DP and DR, connections were observed between DP and both self‐destructive or reckless behavior (E2) and flashback*s* (B3) as well as between trauma‐related amnesia (D1) and both DP and DR (see Figure 2). Given our specific research interest in the connections between DP and DR on the one hand and the other PTSD symptoms on the other hand, Figure 2 visualizes the edge weight estimates for connections between either DP or DR with the other 20 PTSD symptoms. The largest differences between DP and DR were found with respect to their connections to flashbacks and self‐destructive behavior. Additional edge weight difference tests suggested that the edge between DP and self‐destructive behavior was stronger than the edge between DR and self‐destructive behavior and that the edge between DP and flashbacks was stronger than the edge between DR and flashbacks (see Supplementary Materials). However, these findings have to be interpreted with care as the edge weight difference test of the *bootnet* package is not corrected for multiple testing.

In order to further explore the community structure of the network, we performed two community detection analyses following the the helpful suggestion of a reviewer: The spinglass (Newman & Girvan, 2004; Reichardt & Bornholdt, 2006; Traag & Bruggeman, 2008) and walktrap algorithms (Golino & Epskamp, 2017; Pons & Latapy, 2006), currently considered to be the most trustworthy methods (Fried, 2016). These two analyses yielded the finding that, in general, PTSD symptoms appeared to cluster according to the respective *DSM‐5* criteria to which they belonged, with two exceptions. First, the NACM cluster was split into two nodes that reflected negative emotions (D2–D4) and restricted emotions (D5–D7). Second, trauma‐related amnesia (D1) and self‐destructive or reckless behavior (E2) did not cluster with their presumed clusters of NACM and arousal and reactivity reactions, respectively, but instead formed a cluster with DP and DR (see Supplementary Materials for all edge weight differences that were statistically significant in the bootstrapped edge difference tests). Notably, items in the latter cluster were the only items in the dataset that showed a peak at score of 0, and thus these findings should therefore be interpreted with care. For DP and DR, the edge between these two symptoms was significantly stronger than all other edges within the network except for the edges between avoidance of thought (C1) and avoidance of reminders (C2) as well as between hypervigilance (E3) and exaggerated startle response (E4).

### Centrality Measures and Stability

**Figure 3:**
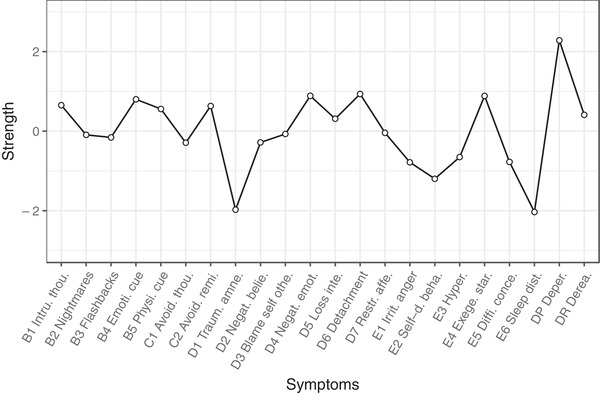
Standardized node strength centrality of the 20 posttraumatic stress disorder (PTSD) symptoms (labels for this group start with either a B, C, D or E, with each letter referring to the respective PTSD criterion in the fifth edition of the *Diagnostic and Statistical Manual of Mental Disorders* [*DSM‐5*]) and two dissociative‐subtype PTSD symptoms (labels are for depersonalization [DP] and derealization [DR]). Intru. thou. = intrusive thoughts; Emoti. cue = emotional cue reactivity; Physi. cue = physical cue reactivity; Avoid. thou. = avoidance of thoughts; Avoid. remi. = avoidance of reminders; Traum. amne. = trauma‐related amnesia; Negat. belie. = Negative beliefs; Blame self othe. = Blame of self or others; Negat. emot. = negative trauma‐related emotions; Loss inte. = loss of interest; Restr. affe. = restricted affect; Irrit. anger = irritability/anger; Self‐d. beha. = self‐destructive/reckless behavior; Hyper. = hypervigilance; Exege. star. = exaggerated startle response; Diff. conce. = difficulty concentrating; Sleep dist. = sleep disturbance.

Our analysis of the stability of the ordering within each centrality measure (i.e., node strength, betweenness, and closeness) revealed that only node strength centrality was stable enough to interpret: correlation–stability coefficient = .671; betweenness and closeness coefficient = .127 (see Supplementary Materials). Figure 3 visualizes the strength centrality estimates, whereby each dot in the figure represents the strength centrality value for each of the 20 PCL‐5 and two D‐PTSD symptoms.

Depersonalization was the most central symptom identified in the network, indicating that it has the strongest connections with other symptoms in the network structure. Strength difference tests revealed that strength centrality of DP was significantly different from all other symptoms except detachment (D6), which was the next most central symptom. However, strength difference tests also revealed that the strength centrality of detachment (D6) was not significantly different from symptoms that were ranked from third through 12th and 14th place, including derealization, which was ranked 10th. Sleep disturbance (E6) was the least central symptom; its strength centrality estimate was significantly smaller than those for all other symptoms except for the other symptoms that ranked in the bottom: trauma‐related amnesia (D1) and self‐destructive or reckless behavior (E2).

To evaluate whether the strong centrality of DP was mainly due to its strong connection with DR, we repeated all analyses with the exclusion of DR. These analyses revealed that DR still ranked as the third‐most central symptom. Moreover, the strength centrality of symptoms was remarkably similar when either the DP or DR items were removed from the analyses (see Supplementary Materials). However, strength difference tests indicated that when DR was excluded, the strength centrality of DP was not significantly different from the strength centrality of symptoms ranked fourth through 12th and 14th (see Supplementary Materials).

## Discussion

In this study, we explored the network connectivity of D‐PTSD symptoms, revealing associations between the experience of DP and DR on the one hand and the reexperiencing, avoidance, NACM, and hyperarousal symptoms of *DSM‐5* PTSD on the other. A number of observations can be made when considering our findings. First, DP and DR exhibited a strong connection, suggesting that these dissociative experiences are highly related to each other. Further, DP emerged as the most central symptom in the network in terms of strength. We note that this result is likely entirely due to the especially strong connection with DR: Connections were also observed with, for example, flashbacks and trauma‐related amnesia, and DP still ranked as the third‐most central symptom after repeating our analyses without DR. However, we cannot rule out that the strong connection between DP and DR is one of the drivers of the strong centrality of DP. Considering our additional analyses without DR, the strength centrality of DP, ranked third, was not significantly different from symptoms that ranked between fourth through 12th and 14th place in terms of their strength. That is, in this sample, without DR, DP was not an exceptionally central symptom. Finally, beyond emphasizing results pertaining to DP and DR, network associations tended to reveal symptom associations broadly consistent with the *DSM‐5* diagnostic clusters. Some of our results are consistent with the results reported in other recent studies (Knefel et al., 2016; McBride et al., 2020), such as findings of a strong connection between DP and DR, and, relatively speaking, a sizeable connection between DP and flashbacks.

A point worth emphasizing is that, whereas Armour, Fried, Deserno, et al. (2017) argue that trauma‐related amnesia might potentially be dropped as a PTSD criterion on the basis that it tended to play no connective role whatsoever in their PTSD networks (but see Greene et al., 2018, in which trauma‐related amnesia was connected to avoidance symptoms in a dynamic network for participants exposed to conflict during the study), we found that although it was generally a peripheral symptom, the inclusion of the D‐PTSD symptoms did seem to create some connectivity of trauma‐related amnesia within the network structure. In particular, trauma‐related amnesia may be more strongly connected with DP and DR as a dissociative experience. Such a finding can be anticipated from experimental results showing that encoding conditions akin to out‐of‐body experiences (i.e., DP), evoked through the use of head‐mounted displays, produced poorer recall for life events in healthy participants and an alteration of left hippocampal response at retrieval (Bergouignan, Nyberg, & Ehrsson, 2014). However, these associations between trauma‐related amnesia and D‐PTSD symptoms in particular need to be interpreted with caution as these symptoms were most frequently not endorsed (i.e., distributions heavily skewed to the right). Although less endorsement of trauma‐related dissociative symptoms in comparison with nondissociative symptoms is predicted on theoretical grounds (Frewen & Lanius, 2014, 2015), we cannot rule out the possibility that a strong association between such skewed symptoms may be, in fact, due to strongly correlated zeroes, which does not necessarily translate into the existence of an actual direct relation between these symptoms. Therefore, the finding of symptom connectivity between DP and DR and trauma‐related amnesia will require replication in other clinical samples, but it serves as an important example of how results of statistical analyses may change depending on which variables are included in these analyses.

Notes of caution are in order, however, regarding the interpretation of cross‐sectional network structures, particularly the extent to which they reflect (a) the presence of causal relations and (b) actual dynamic processes within individuals. Pertaining to the first point, the methods used in the present analyses paper are vetted methodologies for obtaining results that can be interpreted as conditional independence relations (Borsboom et al., 2017). If two variables are conditionally dependent (i.e., there is an edge between these variables) given all other variables in a network, such a finding is consistent with but not sufficient for concluding that a (causal) association exists at group level (Fried & Cramer, 2017). There are various reasons for this, among others that unmeasured confounders (i.e., not part of the estimated network structure) may explain a strong connection between two variables (i.e., the edge is in fact spurious). Alternatively, two variables may be strongly associated because they are indicators, or measurements, of the same underlying construct. Now, the question is whether the nature of the strong association between DP and DR is reflective of an actual direct relation with unknown directionality (e.g., DP → DR) or a spurious relation because both DP and DR are indicators of the same construct (i.e., dissociative experiences). This latter possibility, if true, could bias the centrality result for DP as its high centrality partly stems from a strong connection with DR. Pertaining to the second point, yet also relevant for the first, one needs at least longitudinal data in order to come close to establishing the presence or absence of a causal relationship. In addition, it is indeed not readily possible to extrapolate group‐level results, as we have presented in this paper, to processes within an individual. That is, a strong connection between DP and flashbacks, amnesia, or self‐destructive behavior at the group‐level, for example, does not necessarily imply that individuals in the sample can also be characterized by such a strong connection. Note that this line of reasoning is not unique to network models but holds for every other group‐level model. It is therefore advisable to interpret group‐level results at the level of the group.

The present study suffered from some limitations. First, tertaining to the use of an online community sample of convenience, we cannot rule out the possibility of random responding in order to get through the questions quickly and collect the payment; however, it is worth nothing that, for ethical reasons, we included a response option that allowed respondents to skip each question, but this response option was never selected. Next, the questionnaire did not contain a comprehensive survey of trauma exposure, only childhood trauma exposure. It is possible that some participants reported high posttraumatic stress symptoms for reasons other than childhood trauma exposure, and we cannot rule out that some participants were never exposed to trauma during any time in their lives. These characteristics of our data impose a limit on the generalizability of our results. Third, despite the fact that our data employed a recognized PCL‐5 cutoff score for a likely diagnosis of PTSD, results may nevertheless differ from those that may be observed in a clinically diagnosed sample. A final limitation has to do with the fact that we included participants based on their sum score on the PCL‐5. Because the items of that same PCL‐5 make up the majority of the nodes in our network structure, this strategy harbors the risk of introducing spurious negative edges in the estimated network (Epskamp & Fried, 2018). As such, we have refrained from interpreting the five weak negative edges in our network. We note that we did not have an alternative strategy for deriving a sample due to the lack of suitable variables.

To conclude, the present study identified symptom connections between DP, DR, and the greater symptomatology of *DSM‐5* PTSD. An especially strong connection was observed between DP and DR, and a particularly strong strength centrality was observed for experiences of DP, where connections were also observed between dissociative experiences and flashbacks, trauma‐related amnesia, and self‐destructive behavior. Although broadly consistent with characterizing a dissociative subtype of PTSD, the current observations await replication in other studies assessing different types of trauma exposure not only in childhood but also in adulthood, such as in veterans and treatment‐seeking samples.
